# Description of three new species of *Automeris* Hübner, 1819 from Colombia and Brazil (Lepidoptera, Saturniidae, Hemileucinae)

**DOI:** 10.3897/zookeys.1031.56035

**Published:** 2021-04-15

**Authors:** Thibaud Decaëns, Frédéric Bénéluz, Liliana Ballesteros-Mejia, Diego Bonilla, Rodolphe Rougerie

**Affiliations:** 1 CEFE, Univ Montpellier, CNRS, EPHE, IRD, Univ Paul Valéry Montpellier 3, Montpellier, France Univ Montpellier Montpellier France; 2 Société entomologique Antilles-Guyane (SEAG), 18, Lotissement Amaryllis, F-97354 Rémire-Montjoly, French Guiana Société entomologique Antilles-Guyane Rémire-Montjoly French Guyana; 3 Institut de Systématique, Evolution, Biodiversité (ISYEB), Muséum national d’Histoire naturelle, CNRS, Sorbonne Université, EPHE, Univ des Antilles, Paris, France Sorbonne Université Paris France; 4 CESAB, Centre de Synthèse et d’Analyse sur la Biodiversité, Montpellier, France Centre de Synthèse et d’Analyse sur la Biodiversité Montpellier France; 5 Grupo de Investigación Diseño, Imagen y Comunicación. Facultad de Creación y Comunicación. Universidad El Bosque, Bogotá, Colombia Universidad El Bosque Bogotá Colombia; 6 Tapuragua Reserva Natural de la sociedad civil, Yopal, Casanare, Colombia Tapuragua Reserva Natural de la sociedad civil Yopal Colombia

**Keywords:** Amazonia, DNA barcoding, integrative taxonomy, Neotropics, new species, Orinoco, wild silkmoths

## Abstract

The Saturniidae is one of the most emblematic families of moths, comprising nearly 3000 species distributed globally. In this study, DNA barcode analysis and comparative morphology were combined to describe three new species within the genus *Automeris*, which is the most diverse genus in the family. *Automeris llaneros* Decaëns, Rougerie & Bonilla, **sp. nov.**, *Automeris mineros* Decaëns, Rougerie & Bonilla, **sp. nov.**, and *Automeris belemensis* Decaëns, Rougerie & Bénéluz, **sp. nov.** are described from the Colombian Orinoco watershed, the Colombian Eastern Cordillera, and the area of endemism of Belém in the Brazilian Amazonia, respectively. They all belong to the *Automeris bilinea* (Walker, 1855) species subgroup, which comprises a number of species that are sometimes difficult to distinguish from each other using morphology alone. Here, the description of these three new species is based on significant differences from their closest relatives, either in terms of wing patterns, genitalia, DNA barcodes or a combination of these features.

## Introduction

The Saturniidae family, popularised as the wild silkmoths, is one of the most emblematic families of moths, because of the giant size, colourful patterns, or tailed hindwings of some of its species. In the latest published checklist (Kitching et al. 2018), as many as 3454 valid species were recognised in eight subfamilies and 180 genera. The diversity of the family is highest in the neotropics, where it is represented by six subfamilies and nearly 2400 species that can be found in a broad range of habitats, including the southmost areas of South America, from sea level to elevations exceeding 4000m. The diversity in habitus of these moths is also extreme: size ranges from small moths a few centimetres in wingspan (e.g., in the genus *Hylesia* Hübner, 1820) to very large ones approaching a wingspan of 20 cm (e.g., in *Arsenura* Duncan, 1841); wing patterns can be cryptic, mimicking leaves (e.g., in *Copaxa* Walker, 1855), can harbour large eyespots (e.g., in *Automeris* Hübner, 1919), or can be aposematic in colour (e.g., in *Citheronia* Hübner, 1919); wings can be rounded (e.g., *Dirphia* Hübner, 1919) or elongated (e.g., in *Syssphinx* Hübner, 1819 and *Ptiloscola* Michener, 1949), or some taxa have spectacular tailed hindwings (e.g., *Copiopteryx* Duncan, 1841).

Within subfamily Hemileucinae, the genus *Automeris* comprises species whose size ranges from small to very large; their main feature is the presence of a large eyespot on the dorsal surface of the hindwings. It is the most diverse genus within the family. In his monograph of the subfamily Hemileucinae, Lemaire (2002) listed 135 species in the genus, which he further organised into nine species groups based on the habitus and the structure of genitalia. Recently, the use of molecular approaches such as DNA barcoding, in addition to morphology, led to a significant increase in the pace of discovery and description of new species. Thus, in the past ten years only, as many as 155 new taxa were described, raising the total number of species in the genus to 313 (Kitching et al. 2018). In this paper, we use a combination of morphological features and molecular data (DNA barcodes) to propose the description of three new species from Colombia and Brazil within the group of *Automeris bilinea* (Walker, 1855), which was defined by Lemaire (2002) as a subgroup within the larger species group of *Automeris illustris* (Walker, 1855).

## Materials and methods

### Specimen collecting

Specimens were collected in the following three localities: from July to August 1999 in the savannah landscapes of the Eastern Plains of Colombia (Meta department, TD and DB leg.); in December 2002 in the Boyacá department, Colombia, in an area of humid Andean forest (1500 m in elevation) with moderate level of forest fragmentation (G. Lecourt and DB leg.); and from April to July 2008 in the state of Pará, Brazil, in an area of Amazonian forest with moderate to high levels of forest fragmentation (TD leg.). Moths were attracted by a Mercury Vapour (MV) bulb powered by a small portable generator. A white sheet of 2 m height × 3 m width was used as a reflector. Collecting took place throughout each entire night, i.e., from 18:30 h to 06:30 h, in order to increase the probability of detecting species with different flight behaviours (Lamarre et al. 2015). Moths coming to the sheet were injected with ammonia, stored, and dried in labelled paper envelopes and brought to the lab to be mounted for morphological examination.

### Morphological descriptions

All the specimens were mounted in a standard way to allow optimal examination of their body and wings. Male genitalia and eighth abdominal segment were prepared in 10% caustic potash solution to remove piliform scales, and were preserved in 75% ethanol. Body morphology, wing ornamentation and male genitalia structure were described using the terminology of Lemaire (1971, 2002).

Morphological features of the prepared specimens were compared with those of the species represented in Lemaire (2002) and Brechlin and Meister (2014). Additionally, type specimens of the three newly described species were compared with specimens from closely related species available in collections of TD and MNHN: 3 ♂ of *Automeris belizonensis* Brechlin & Meister, 2014; 2 ♂♂ and 1 ♀ of *Automeris bilinea* (Walker, 1855); 16 ♂♂ and 1 ♀ of *Automeris cinctistriga* (Felder & Felder, 1874); 5 ♂ of *Automeris fieldi* Lemaire, 1969; 5 ♂♂ of *Automeris godartii* (Boisduval, 1875); 3 ♂♂ of *Automeris lemensis* Lemaire, 1972; 28 ♂♂ and 1 ♀ of *Automeris midea* (Maassen & Weyding, 1885).

### DNA barcoding and molecular analyses

DNA was extracted from dry legs removed from dry collection specimens of the suspected new species. We sampled two specimens of *Automeris llaneros* sp. nov., five specimens of *Automeris mineros* sp. nov. and 17 specimens of *Automeris belemensis* sp. nov., and we also included sequences of closely related species obtained from the Barcode of Life Data Systems (BOLD; Ratnasingham and Hebert 2007) and that had been generated as part of the DNA barcoding campaign for saturniid moths, coordinated by RR. Tissue samples were processed at the Canadian Centre for DNA Barcoding (CCDB). DNA was extracted using a routine silica-based 96-well extraction automation protocol (Ivanova et al. 2006). The part of COI used as a ‘DNA barcode’ (Hebert et al. 2003) was amplified with the primer set LepF1/LepR1 (Hebert et al. 2004), targeting a 658 bp fragment. The DNA extracts that did not amplify for the full-length DNA barcode were re-amplified with the internal primer pairs LepF1/MLepR1 and MLepF1/LepR1, targeting DNA fragments of 307 bp and 407 bp (Hajibabaei et al. 2006), respectively. All PCR amplifications were performed according to the standard PCR reaction protocol used in CCDB (Hajibabaei et al. 2005); PCR products were checked on a 2% E-gel 96 Agarose (Invitrogen, Burlington, ON, Canada). Unpurified PCR fragments were sequenced in both directions using the same primers as for the PCR reaction. The sequencing reactions followed CCDB protocols (http://ccdb.ca/resources/; Hajibabaei et al. 2005). All sequences were aligned and inspected for frame-shifts and stop codons for removal of editing errors and possible pseudogenes.

All records, including specimen and sequence data, and GenBank accession numbers, are given in Appendix 1, and are publicly accessible in the Barcode of Life Data system (BOLD) within dataset DS-AUTONSP (https://doi.org/10.5883/DS-AUTONSP). An unrooted neighbour joining (NJ) tree was computed on BOLD V4 using p-distances and the BOLD aligner option to compare the sequences obtained from the specimens of the three new species and those of closely related taxa. BOLD was used to calculate uncorrected p-distances between newly described species and their closest relatives. We also used the barcode identification numbers (BINs), i.e., clusters of barcode sequences automatically generated in BOLD which have a high concordance with species, as an additional source of information for species discrimination (Ratnasingham and Hebert 2013).

### Distribution maps

We present maps of the current distribution for the three newly described species and their closest species within the *A. bilinea* subgroup (seven species, Fig. 3). Records of the other species within the *A. bilinea* subgroup were collected from BOLD and were georeferenced and carefully curated for locality and species identity excluding any possible error (a complete list of specimens and associated data is given in Appendix 1).

### Collection abbreviations

**CCGM** collection of Carlos G. Mielke (Ponta Grossa, Brazil);

**CDB** collection of Diego Bonilla (Yopal, Colombia);

**CFB** collection of Frédéric Bénéluz (Matoury, French Guiana);

**CTD** collection of Thibaud Decaëns (Montpellier, France);

**IAvH** Instituto de Investigación de Recursos Biológicos Alexander von Humboldt (Bogotá, Colombia);

**MNHN**Muséum national d’Histoire naturelle of Paris (France);

**MPEG**Museu Paraense Emilio Goeldi (Belém, Brazil).

## Taxonomic account

### Automeris mineros

Taxon classificationAnimaliaLepidopteraSaturniidae

Decaëns, Rougerie & Bonilla sp. nov.

E69CFE8B-3FA2-5408-83CC-7352B315C882

http://zoobank.org/CE1EAA15-8197-4B98-A2F4-062C40A83153

**Figure 1.**
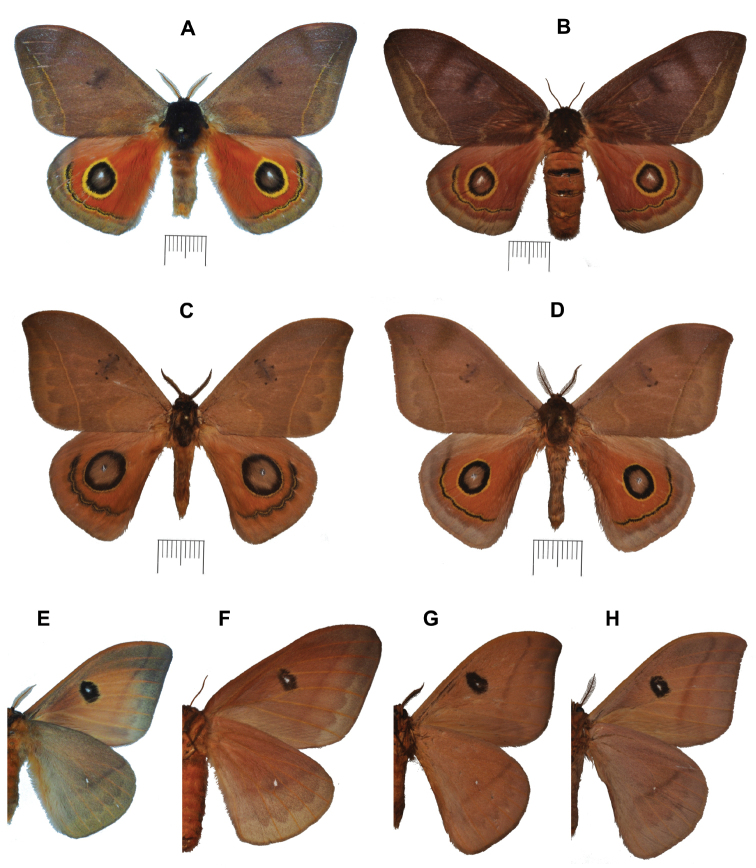
Specimens of the new species of *Automeris* spp. **A** dorsal view of *A. mineros* sp. nov., holotype ♂ **B** dorsal view of *A. mineros* sp. nov., paratype (allotype) ♀ **C** dorsal view of *A. belemensis* sp. nov., holotype ♂ **D** dorsal view of *A. llaneros* sp. nov., holotype ♂ **E** ventral view of *A. mineros* sp. nov., holotype ♂ **F** Ventral view of *A. mineros* sp. nov., paratype (allotype) ♀ **G** ventral view of *A. belemensis* sp. nov., holotype ♂ **H** ventral view of *A. llaneros* sp. nov., holotype ♂. Scale bars: 1 cm.

**Figure 2.**
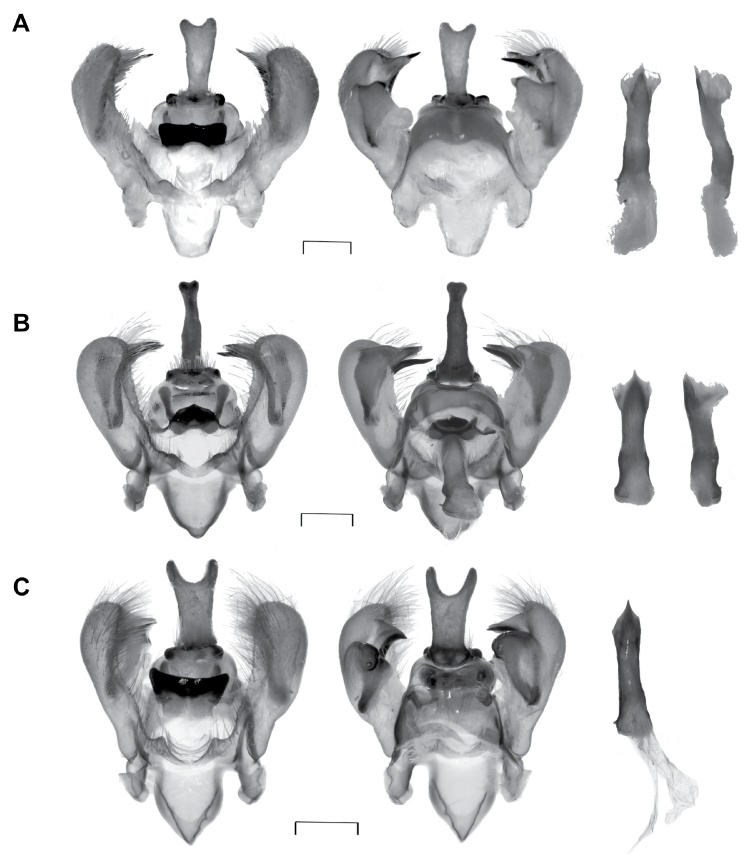
Genitalia ♂ of the new species of *Automeris* spp. **A***A. mineros* sp. nov., paratype ♂ (BC-Dec0549) **B***A. belemensis* sp. nov., paratype ♂ (BC-INCT1136) **C***A. llaneros* sp. nov., paratype ♂ (BC-Dec0712). For each species, the dorsal and ventral views of the genitalia, and the dorsal and lateral views of the aedeagus are represented from the left to the right. Scale bars: 1 mm.

Figures 1A, B, E, F , 2A

#### Type material.

***Holotype*.** Colombia • ♂ (Fig. 1A, E); Boyacá department, near Quipama, Vereda Caviche; 5.575°N, 74.2595°W; elevation: 1500 m; 1–3 Dec. 2002; at MV light; DB and G. Lecourt leg.; BOLD SampleID: BC-Dec0551; Deposited in IAvH.

***Paratypes*** (19 ♂♂ and 6 ♀♀). Colombia • 15 ♂♂ and 5 ♀♀, all same data as holotype; all specimens collected at MV light except one pair *ab ovo*, reared on *Pyracantha regersiana* in Rouen (France) by TD, and 3 ♂♂ and 2 ♀♀ ab ovo, reared on *Quercus* sp. in Bogotá (Colombia) by L.D. Ramirez and DB. Deposited as follow: 2 ♂♂ and 2 ♀ (Fig. 1B, F; allotype; BOLD SampleID: BC-Dec0547) in IAvH, 5 ♂♂ in MNHN (BOLD SampleID: BC-Dec0548, BC-Dec0549, BC-Dec0550), 6 ♂♂ and 1 ♀ in CTD, 1 ♂ in CFB, 4 ♂♂ and 2 ♀♀ in CCGM, 1 ♂ and 1 ♀ in CDB.

#### Diagnosis.

**Figure 3.**
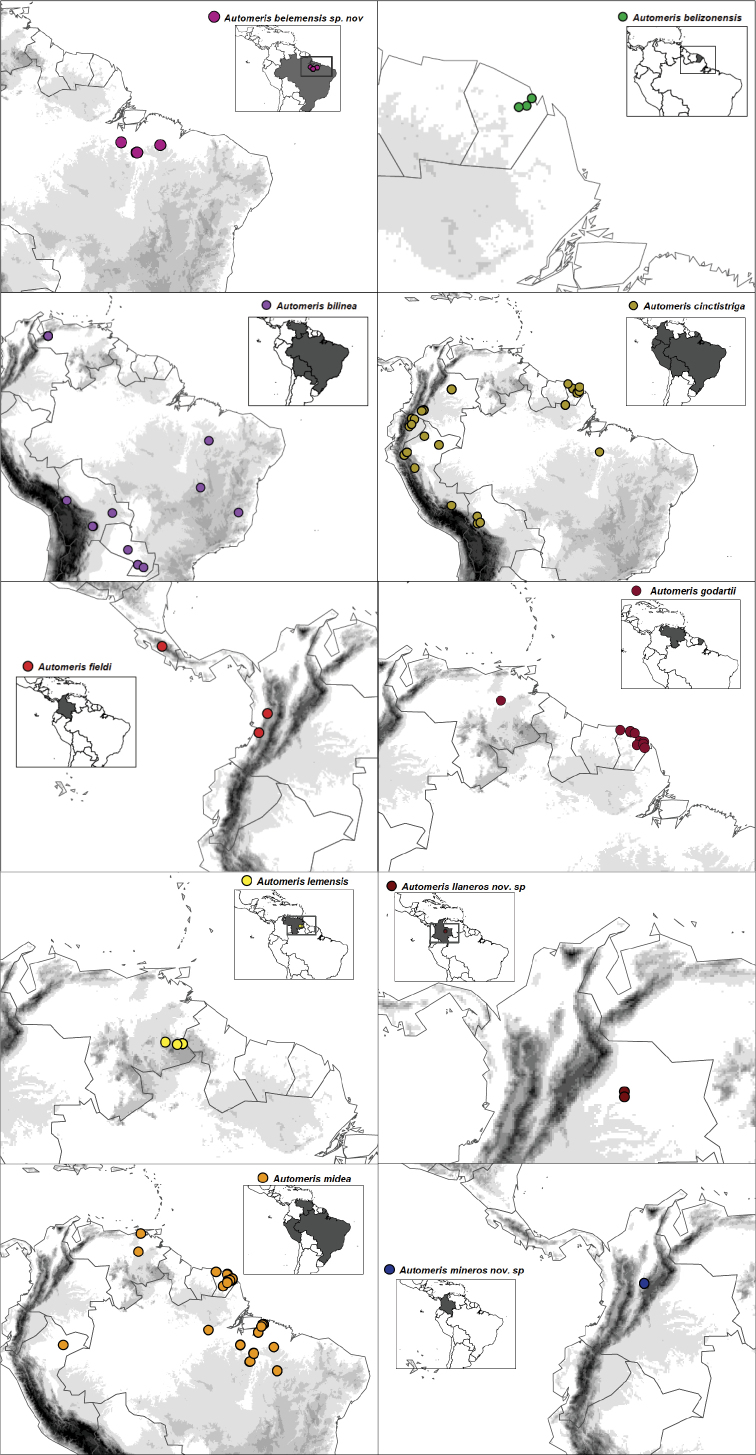
Distribution maps of *Automeris* species within the subgroup of *A. bilinea*, based on georeferenced records collected from BOLD, and known geographic distribution of the three new species of *Automeris* spp.

**Figure 4.**
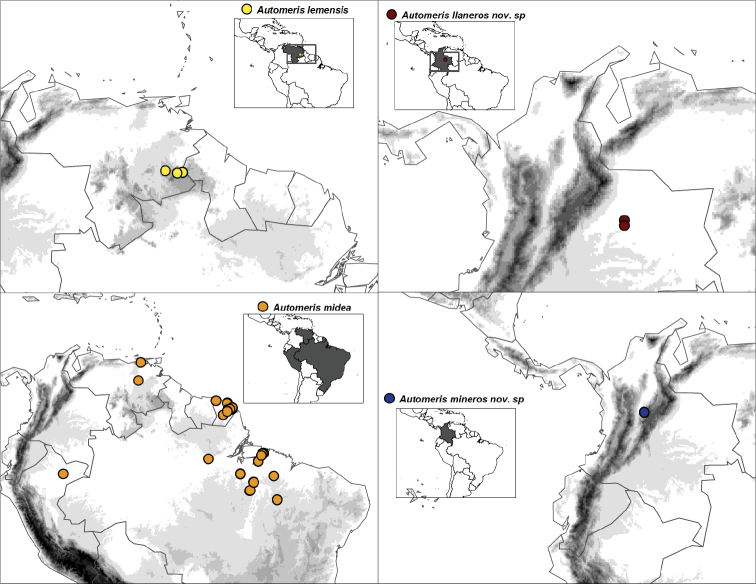
Neighbour joining tree (K2P distances) built from DNA barcodes (COI) of specimens from the three new species of *Automeris* and their closest relatives. The labels of the terminal branches successively give the following information: sample ID code in the Barcode of Life Data system, country, and exact collecting site when available, Barcode Identification Number (BIN) automatically assigned to each sequence in BOLD. All specimens of *A. lemensis* and one of *A. bilinea* have no BIN number due to the short length of their COI sequences.

*Automeris mineros* sp. nov. is similar to the reddish forms of *A. midea*, a species with a large and essentially Amazonian distribution (Fig. 3). However, the vivid coloration, which is occasional in the later, is consistent among all the specimens of *A. mineros* sp. nov. that have been examined. It is also possible to separate the two species by additional fine characters of the habitus. In *A. mineros* sp. nov., the antemedian area of the forewings appears lighter than the median area due to the presence of a dense dusting of yellow scales, while this zone is generally concolourous with or darker than the rest of the wing in *A. midea* (Fig. 4). The apex of the forewing tends to be less acute in *A. mineros* sp. nov. The width of the yellow periocellar ring of the hindwing is also wider in *A. mineros* than in most of the examined specimens of *A. midea*. Finally, the veins on the ventral side of the forewings are clearly highlighted in orange in *A. mineros* sp. nov., and are more sharply contrasting with the surrounding ground colour than in *A. midea*. Distinction based on male genitalia is less conclusive, although we can however note the truncated instead of triangular shape of the saccus, as well as the median plate of the gnathos which is less massive in *A. mineros* sp. nov. and clearly marked by a median projection. Interestingly, DNA barcodes clearly separate *A. mineros* sp. nov. from all closely related species in a distinct cluster of sequences (BINBOLD:ABY4503; see fig. 4). The nearest neighbour is *A. belizonensis* (1.8% minimum p-distance), from which it can be distinguished by the more rounded shape of the forewings and the more vivid coloration of the hindwings.

#### Description.

♂ (Fig. 1A, E). ***Wingspan***: 77–84 mm. ***Head***: dark brown, labial palpi and antennae orange brown. ***Thorax***: dorsally dark brown with red orange piliform scales on the ventral side; legs dark brown. ***Abdomen***: dark orange brown with dark brown piliform scales on the dorsal side; eighth abdominal segment lacking any remarkable sclerotised structure. ***Forewings***: length 40–42 mm, slightly elongated, rounded apex, straight outer margin; dorsal ground colour orange brown, suffused with yellow scales in the ante- and postmedian areas and, to a lesser extent, by pink scales in the median area; antemedial line faint, almost indistinct, only visible as yellow scales bordering its distal edge; postmedial line barely preapical (1–3 mm), slightly convex from apex to vein CuA2, then bent toward the anal margin, yellow in colour, lined distally by a line of black scales; discocellular mark rectangular, darker than the surrounding wing surface, with a dark brown spot in its centre, and three to four small spots of the same colour at its corners. Ventral side with a large dull orange area extending on the main basomedian area, with veins marked in distinctive orange scales; apical area dark brown, extending along outer margin and toward tornus. Postmedial line well marked, dark reddish brown; marginal band diffuse, suffused with yellow scales and disappearing toward apex. Discocellular mark large and black, with a white discal spot in its centre and surrounded by a thin diffuse ring of yellow scales. ***Hindwings***: basomedian area vivid orange-red with a 7–12 mm × 6–8 mm eyespot in its centre formed by, from its centre: a small well marked white pupil surrounded by a dark brown iris, a first large black periocellar ring, a second large yellow ring of the same width, and finally a barely visible line of black scales enclosing the eyespot. Postmedial line black and lunular, distally bordered by a thin line of yellow scales, and proximally by another thin line of yellow and black scales; postmedian area formed by a thin vivid orange-red band and bordered by a large orange-brown marginal band covered with yellow scales. Ventral side light brown, suffused with yellow scales, particularly on the marginal band; venation distally marked with orange scales. Postmedial line dark brown, becoming faint toward the anal margin; discal cell with a small white spot.

***Wingspan*** ♀ (Fig. 1B, F): 94 mm. ***Head, thorax, and abdomen*** of the same colour as in the male. ***Forewings***: length 47 mm, elongated, rounded apex, almost straight border; dorsal ground colour dark purplish brown, suffused with light grey scales in the median area, except for a large oblique band lacking these scales approximately half way between the dark rectangular discal mark and the apex; postmedian area suffused with yellow scales. Both ante- and postmedial lines yellow, the latter straight and slightly preapical (3 mm). Ventral side light orange brown, the venation marked by orange scales, marginal band suffused with orange scales; postmedial line dark brown; large black discocellular mark, with a large white discal spot in its centre. ***Hindwings***: Basomedian area brownish red; eyespot with the same structure as in the male, slightly duller in colour; black and lunular postmedial line, bordered by a line of yellow scales on both edges; postmedian area brownish red; marginal band covered with yellow scales. Ventral side light brown, suffused with yellow to pink scales; venation distally marked by orange scales; discal point small and white.

***Genitalia*** ♂ (Fig. 2A): typical of the *A. bilinea* subgroup as described in Lemaire (2002). Uncus well developed, slightly extending beyond the distal end of the valves, with a broad bifid dorsal protuberance. Dorsal lobes of valves weakly developed and sharp. Median plate of gnathos strongly sclerotised with its posterior margin concave and with a small median projection. Saccus short and anteriorly truncated. Phallus straight, with a small lateral spine on its base; its posterior, tapering end slightly bent upward, with the weakly developed vesica expanding ventrally.

***Genitalia*** ♀: not examined.

#### Immature stages.

Eggs were obtained from a wild collected female. Larvae hatched 22 days after and readily fed on *Pyracantha regersiana* (Rosaceae) in France (rearing #17 by TD) and on *Quercus* sp. (Fagaceae) in Colombia (rearing by L. D. Ramirez and DB). Native foodplants remain unknown. Rearing was successful in plastic boxes, feeding larvae with fresh branches changed every 2–4 days. Larvae completed six instars within two months on *P. regersiana* and pupated in a brown cocoon.

***Eggs*** are white with a black micropyle, laterally flattened, 2 mm diameter × 0.8 mm height, laid in dense cluster of several dozens. ***First larval instar***: head black. Body 4 mm upon hatching, 6 mm maximal length; pale yellow with black scoli and spines. ***Second instar***: Head black. Body 7–8 mm maximal length; brownish yellow dorsally, dull yellow ventrally; scoli and spines dark brown. ***Third instar***: Head black. Body: 14 mm maximal length; brownish yellow dorsally, green yellow ventrally; scoli and spines dark brown. ***Fourth instar***: Head black. Body 19–20 mm maximal length; dark brown dorsally with fine light green stripes, light green ventrally; scoli and spines dark brown to black. ***Fifth instar***: Head green. Body: 35–40 mm maximal length; light green colour with pink dorsal ornamentation, a lateral ivory strip ventrally and dorsally bordered with a thin black line; scoli and spines light green. ***Sixth instar***: Same colour and ornamentation as previous instar; 35–40 mm maximal length. ***Pupa and cocoon***: Last instar larvae spin a thin and supple cocoon of beige silk. Pupa 24–37 mm long, dark brown. Reared adults emerged from the cocoon early in the morning one to two months after pupation.

#### Distribution.

*Automeris mineros* sp. nov. is known form the type locality only, in the Oriental Cordillera of Colombia near Muzo (Fig. 3), a region from which a number of new taxa of Saturniidae were described recently (Decaëns and Rougerie 2008).

#### Etymology.

This species is named in reference to emerald mining, which represents an emblematic economic activity in the region surrounding the type locality.

### Automeris belemensis

Taxon classificationAnimaliaLepidopteraSaturniidae

Decaëns, Rougerie & Bénéluz sp. nov.

28FDABE2-62FC-5983-B736-626A8F777467

http://zoobank.org/A1CECFCF-0A6B-4128-B4FE-673D66FB6255

Figures 1C, G , 2B

#### Type material.

***Holotype*.** Brazil • ♂ (Fig. 1C, G); Pará state, Maçaranduba, Nova Ipixuna; Apr. 2008; 4.7990°S, 49.3630°W; elevation: 100 m; at MV light; TD leg.; BOLD SampleID: BC-TDMPEG0008; deposited in MPEG (catalogue number: MPEG.HLE 04018743).

***Paratypes*** (16 ♂♂). Brazil • 13 ♂♂, same data as holotype with different sampling locations in the same area: 4.7990°S, 49.3630°W; 4.8110°S, 49.3670°W; 4.8050°S, 49.3690°W; 4.8040°S, 49.3230°W. Brazil • 1 ♂; Pará state, Pacajá; June 2008; 3.7060°S, 51.0390°W; at MV light; TD leg. Brazil • 2 ♂♂; Maranhão state, Reserva Biologica do Gurupi; 18 Apr. 2010; 4.0014°S, 46.8372°W; at MV light; TD leg. Deposited as follow: 3 ♂♂ in MPEG (BOLD SampleID: BC-TDMPEG0667, BC-TDMPEG0743, BC-TDMPEG0744; MPEG catalogue number: MPEG.HLE 04018744, MPEG.HLE 04018745, MPEG.HLE 04018746), 4 ♂♂ in the MNHN (BOLD SampleID: BC-TDMPEG0918, BC-TDMPEG0919, BC-INCT1136, BC-INCT1137), 3 ♂♂ in CFB (BOLD SampleID: BC-TDMPEG0956, BC-TDMPEG0957, BC-TDMPEG0982), 2 ♂♂ in CCGM (BOLD SampleID: BC-TDMPEG0983, BC-TDMPEG0920), 4 ♂♂ in CTD (BOLD SampleID: BC-TDMPEG0007, BC-TDMPEG0009, BC-TDMPEG0014, BC-TDMPEG0301).

#### Diagnosis.

Phenotypically, *Automeris belemensis* sp. nov. is closely related to *A. cinctistriga* and *A. godartii* from which it is difficult to separate based on wing patterns, particularly if we consider the phenotypic variability that characterises these species (see Fig. 3 for distribution map of these species). However, the examination of a large number of specimens reveals subtle characters that make it possible to differentiate them. The distinction from *A. cinctistriga* is possible because of the general shape of the forewings in *A. belemensis* sp. nov. usually less elongated with a less prominent apex, and by the ornamentation of the eyespot of the hindwings, whose yellow ring is thinner, and highlighted externally by the presence of a line of black scales which is usually lacking in *A. cinctistriga*. The distinction from *A. godartii* is more difficult, but the general colouration of the wings is, however, different in the 22 specimens examined of the two species. In *A. belemensis* sp. nov., the ground colour of the forewings is a lighter orange-brown than the grey-brown ground colour that is characteristic of *A. godartii*. The dusting of silver scales generally present in *A. godartii*, in particular in the preapical triangle of the forewings, is weakly marked or completely absent in *A. belemensis* sp. nov. The ante- and postmedial lines are also lighter in *A. belemensis*, standing out in a more contrasted way compared to the surrounding wing colour. Finally, the line of black scales surrounding the eyespot of the hindwings, and sometimes even the yellow periocellar ring, which are continuous in *A. godartii*, are frequently interrupted towards the subcostal area in *A. belemensis* sp. nov. It is likely that the male of *A. godartii* figured in Lemaire (2002: plate 40, fig. 4), originating from the state of Pará in Brazil, actually belongs to *A. belemensis* sp. nov.

With a long posteriorly produced uncus, male genitalia are similar to those of *A. godartii*, but also to those of *A. lemensis*, which is known only from the Gran Sabana region in southern Venezuela (Fig. 3). Interestingly, the DNA barcodes of the 11 sequenced specimens of *A. belemensis* sp. nov. form a distinct cluster in the NJ tree (BINBOLD:AAA5242) with *A. lemensis* as the nearest neighbour with 2% minimum uncorrected p-distance (Fig. 4). However, both species can easily be distinguished by the ground coloration of their wings, which is much duller in the former, especially in the periocellar area of the hindwings, and an otherwise much rounder hindwing eyespot.

#### Description.

♂ (Fig. 1C, G). ***Wingspan***: 70–71 mm. ***Head***: dark brown, labial palpi and antennae orange brown. ***Thorax***: dorsally dark brown and ventrally orange brown; legs dark brown. ***Abdomen***: orange brown. ***Forewings***: Length 35–37 mm, slightly elongated, slightly prominent apex, straight outer border; dorsal ground colour orange brown; ante- and postmedial lines thin and yellow, the latter slightly convex, becoming barely visible upon reaching the costal margin 3–4 mm from the apex; discocellular mark rectangular, darker than the surrounding wing surface, with four to six dark brown spots at its angles. Ventral side yellow brown, slightly darker on the outer margin, with the postmedial line underlined by dark brown scales, and a large black discocellular spot, marked in its centre by a small white discal dot. ***Hindwings***: basomedian area dull orange with a large eyespot in its centre formed by, from its centre: a small black and white pupil, a large grey brown iris surrounded by a broad black periocellar ring, followed by a thin yellow ring, and finally by an external line of black scales. Postmedial line lunular, formed by a thin yellow line bordered proximally and distally by a wider black line; postmedian area dull orange; marginal band orange brown. Ventral side uniformly yellow brown, with a straight, weakly marked postmedial line, a faint zigzagging premarginal line, and with a small white discal spot.

**Female** unknown.

***Genitalia*** ♂ (Fig. 2B): similar general structure as in other species of the *bilinea* subgroup. Uncus well developed, long and bent downwards, apically barely bifid. Dorsal lobes of valves well developed and broadly triangular. Median plate of gnathos strongly sclerotised with its posterior margin convex, a developed median projection, and long lateral appendages. Saccus well developed, triangular and acute anteriorly. Phallus straight, with a small lateral spine on its base; its posterior end slightly bent upward, with a weakly developed vesica.

#### Distribution.

*Automeris belemensis* sp. nov. is known from the lower Amazonian watershed in the Brazilian states of Pará and Maranhão, Brazil (Fig. 3). It has not been found despite of extensive collecting efforts in neighbouring region of French Guiana, and is also unknown from other areas of Brazilian Amazonia. This suggests it could be restricted to the Belém area of endemism as defined in Cardoso da Silva et al. (2005), which was recently highlighted as a hotspot for the diversity of so far undescribed moth species (Lamarre et al. 2016).

#### Etymology.

*Automeris belemensis* sp. nov. is named as a reference to the area of endemism of Belém where this species has been found and to which it is likely endemic.

### Automeris llaneros

Taxon classificationAnimaliaLepidopteraSaturniidae

Decaëns, Rougerie & Bonilla sp. nov.

E8735EB1-0D5C-508E-A5C2-1B4159BF150F

http://zoobank.org/790F11B2-06F1-4CCF-9AA1-423401D11432

Figures 1D, H , 2C

#### Type material.

***Holotype*.** Colombia • ♂ (Fig. 1D, H); Casanare, Orocue; 4.7943°N, 71.3353°W; elevation: 150 m; Aug. 1999; at MV light; TD and DB leg.; BOLD SampleID: BC-Dec0711; deposited in the IAvH.

***Paratype*.** Colombia • 1 ♂; Meta, Carimagua research station; 4.5716°N, 71.3320°W; elevation: 170 m; July 1999; at MV light; TD and DB leg.; BOLD SampleID: BC-Dec0712; deposited in CTD.

#### Diagnosis.

*Automeris llaneros* sp. nov. is phenotypically very similar to *A. cinctistriga*, from which it is quite difficult to distinguish based on wing patterns alone. However, the two known specimens of *A. llaneros* sp. nov. have less elongated forewings with less acute apices than most of the examined specimens of *A. cinctistriga*. The background colour of the forewing is also duller in *A. llaneros* sp. nov., less orange, and the ante- and postmedian lines are finer, beige instead of yellow, and contrasting much less markedly with the general colour of the wings. Finally, the distance between the ante- and postmedian lines at the point where they join the anal edge of the forewings seems greater in *A. llaneros* sp. nov. (1 cm in the two known specimens) than in *A. cinctistriga* (4–7 mm). The DNA barcodes of *A. llaneros* sp. nov. are assigned to a different BIN than those of *A. cinctistriga* (see discussion), and the two species are very clearly separated in the DNA barcode tree, bringing additional support to their treatment as two distinct species.

DNA barcodes place *A. llaneros* sp. nov. near *A. belizonensis*, *A. mineros* sp. nov., and *A. fieldi* on the NJ tree (Fig. 4). This proximity seems to be confirmed by the examination of the male genitalia, whose bifid protuberance of the uncus is strongly developed, exceeding the tip of the valves, as in *A. mineros* sp. nov. (described here), *A. belizonensis*, and *A. fieldi* (Lemaire 1971; Brechlin and Meister 2014). In comparison, *A. cinctistriga* generally presents a less developed and less deeply indented uncus (Lemaire 1971). The new species is also easily distinguished from these close relatives based on its wing shape and patterns. For instance, *A. belizonensis* has more elongated forewings with more pointed apex, and an overall more vivid and orange colouring. *Automeris mineros* sp. nov. also has a very different coloration, notably due to the contrasting orange-red periocellar area of the hindwings. Finally, *A. fieldi*, a species occurring from the Pacific slopes of the Andes to Costa Rica (Fig. 3) and probably north to Honduras (Bénéluz, pers. comm.), stands out again by the slightly more elongated shape of the forewings, but also by the presence of a continuous ring of black scales external to the eyespot of the hindwings, which is lacking in *A. llaneros* sp. nov.

#### Description.

♂ (Fig. 1D, H). ***Wingspan***: 72–74 mm. ***Head***: Dark brown, labial palpi and antennae brown. ***Thorax***: dorsally dark brown and ventrally light brown; legs light brown. ***Abdomen***: dorsally orange brown, ventrally light brown. ***Forewings***: Length 32–35 mm, slightly elongated, slightly prominent apex, straight to slightly convex outer border; dorsal ground colour dull orange brown; antemedial line thin and beige, doubled with a brown line proximally; postmedial line slightly convex, barely visible as it reaches the costal margin 2–3 mm from the apex, thin and beige, distally bordered by dark brown scales; basal and median areas concolorous; discocellular mark of the median area rectangular, darker than the surrounding wing surface, with three clearly visible dark brown spots at corners and one faint central spot. Ventral side yellow brown, with darker postmedial and premarginal lines, and a large black discocellular mark, marked in its centre by a white discal spot; venation marked with orange scales. ***Hindwings***: Basomedian area dull orange suffused by dark brown scales, with a large eyespot in its centre formed by, from its centre: an almost completely white small pupil, suffused with few black scales, a large grey brown iris, surrounded by a large black ring and then a thinner yellow ring (in paratype specimen we observed a few black scales external to this outer yellow ring of the eyespot); postmedial line black, lunular, bordered proximally and distally by thin lines of yellow scales; postmedian area dull orange; marginal band large and grey. Ventral side uniformly orange brown, with a weak oblique postmedial line interrupted before it reaches the costal margin, a vestigial premarginal line forming darker U-shaped marks between veins, and a small white discal point.

**Female** unknown.

***Genitalia*** ♂ (Fig. 2C): very similar to those of *A. mineros* sp. nov. Uncus elongated, large and strongly bifid apically, largely extending beyond the valves. Valves relatively short, rounded, with a broad, rounded dorsal lobe; arms short and strongly curved. Median plate of the gnathos highly sclerotised, as wide as the saccus and subrectangular. Saccus well developed, triangular and acute anteriorly.

#### Distribution.

*Automeris llaneros* sp. nov. is only known from the region of Carimagua and Orocué, in the Colombian part of the Orinoco watershed, the so called “Llanos Orientales” of Colombian Eastern Plains (Fig. 3). The region has been poorly investigated for saturniid diversity but is known to host a few endemics whose exact distributions need to be clarified (see for example Decaëns et al. 2005).

#### Etymology.

This species is named in reference to the Llanos region, which refer to the large area of savannahs that cover most of the Colombian and Venezuelan Orinoco watershed.

## Discussion

The description of three new species of *Automeris* within the highly cryptic *A. bilinea* species-subgroup is a new illustration of the value of DNA barcoding, when combined with morphological diagnostic characters and distribution data, in disclosing hidden diversity (Hebert et al. 2004). Our study illustrates two different methodological approaches, which can allow the identification and support the description of new species in such taxonomically difficult groups.

In the case of *A. mineros* sp. nov., the species had appeared clearly distinct from other known species by the study of its external habitus, showing in particular a unique coloration of the hindwing periocellar area. This singularity is however shared with some specimens representing extreme variants of another species belonging to this subgroup (*A. midea*). This led us to further explore the question, first by a comparison of male genitalia, used conventionally but whose discriminating characters between closely related species in this group sometimes remains uncertain or even equivocal (Lemaire 1971). Finally, we used DNA barcodes as an independent dataset to confirm the separation of *A. mineros* sp. nov. from its relatives.

For *A. llaneros* sp. nov. and *A. belemensis* sp. nov., the approach was different. The use of DNA barcodes first revealed the existence of cryptic species, i.e., species presenting different barcodes but that were not discriminable from their habitus, or differing from each other only by subtle characters. The subsequent analysis of the genetic distances revealed affinities with species which were not necessarily the most similar in their wing pattern, but such affinities were corroborated in both cases by the structure of the male genitalia. Thus, it is the combination of the genitalia, the habitus, and the DNA barcodes which concomitantly characterise these species within the group.

In the case of *A. llaneros* sp. nov., the problem of the real identity of *A. cinctistriga*, a species with which it is impossible to rule out confusion, also deserves to be considered carefully. *Automeris cinctistriga* was described from a male collected in Colombia, and Lemaire (1971) already stated that the exact identity of this species could be problematic, as the abdomen of the lectotype had been destroyed. It is also most likely that the type-locality of the lectotype, Bogotá (2600m asl), is wrong, as no similar species seems to fly at such high elevation Andean forests of the Colombian Eastern cordillera (pers. obs.). Within the material collected in the eastern plains of Colombia, we found several specimens corresponding to the description of *A. cinctistriga* based on their wing patterns, and DNA barcoding of these specimens revealed two distinct clusters of barcodes corresponding to two distinct BINs in BOLD. These two BINs were clearly separated in the barcode tree (Fig. 4), making *A. cinctistriga* in its former definition paraphyletic, and clearly suggesting that they correspond to two different species (Mutanen et al. 2016). In the absence of genitalia and available DNA sequences from the old lectotype of *A. cinctistriga*, it was formally impossible to define which of the two species corresponded to each of these BINs. We therefore adopted the more conservative position. We considered that *A. cinctistriga* corresponded to the BINBOLD:AAA8445, which in BOLD, is defined by a large distribution around the Amazonian watershed (including Bolivia, Brazil, Colombia, Ecuador, French Guiana, Peru), perfectly fitting the distribution of *A. cinctistriga* as described in the literature (Lemaire 1971, 2002). The other BIN (BOLD:ABZ3239) was represented by the two Colombian specimens used herein as type material for the description of *A. llaneros* sp. nov. This position is also consistent with wing ornamentation, since the lectotype represented by Lemaire (1971) broadly presents the characteristics that we have attributed to *A. cinctistriga*.

Overall, the discovery of new species, including some cryptic ones, in a highly diverse genus such as *Automeris* does not represent a surprising finding. The cryptic diversity of *Automeris* has already been highlighted by recent taxonomic studies, in which traditionally recognised species with large geographical distributions have been divided into several new species based primarily on differences in DNA barcodes (Brechlin and Meister 2014). This underlines the extent of the taxonomic deficit that characterises the family Saturniidae, which nevertheless is among the best studied within the “Heterocera”.

The fact that species traditionally recognised as having a wide distribution prove to be in fact complexes of cryptic species with more restricted distributions also raises new and interesting questions concerning the specificity of the different biogeographical areas which constitute the neotropics. The three regions from which the species described in our study originate are a perfect example. The saturniid fauna is considered to be made up of a mixture of endemics and widely distributed species, with variable proportions depending on the regions. For instance, the Eastern Cordillera of Colombia is considered to be a hotspot of diversity, harbouring a significant proportion of endemics (Lemaire 2002; Decaëns et al. 2007; Decaëns and Rougerie 2008), whereas faunas of the Orinoco and Amazon lowlands are generally considered to be dominated by species with wide distributions (Lemaire 2002). In all cases, however, it is likely that due to this cryptic diversity, a significant proportion of species diversity has been underestimated by the predominant use of morphology in previous estimations. This is well illustrated by our case study, where we describe three new species with probably restricted distributions based largely on the information provided by DNA barcoding. As described in other groups of organisms (Guarnizo et al. 2015), we can therefore expect that the generalisation of the use of DNA barcoding will continue to fragment the expansive distributions of widespread species, thus modifying our current perception of diversity distributed among biogeographical areas.

## Supplementary Material

XML Treatment for Automeris mineros

XML Treatment for Automeris belemensis

XML Treatment for Automeris llaneros

## Appendix 1

**Table:** List of the specimens included in the dataset DS-AUTONSP of the Baorcode of Life Data system, collecting data and accession codes in BOLD and GenBank.

Species	Sex	Status	Collectors	Collection Date	Country	State/Province	Exact Site	Latitude / Longitude	Elevation (m)	Collection Date	Collectors	COI-5P Seq. Length	Sample id	Process ID	BIN	GenBank accession
*Automeris belemensis* sp. nov.	♂	Holotype	T. Decaens	01-Apr-2008	Brazil	Para	Macaranduba	-4.7990, -49.3630	102	01-Apr-2008	T. Decaens	658[0n]	BC-TDMPEG0007	TDMPE007-09	BOLD:AAA5242	JN827467
	♂	Paratype	T. Decaens	18-Apr-2010	Brazil	Maranhao	Acailandia, REBIO do Gurupi	-4.0014, -46.8372	275	18-Apr-2010	T. Decaens	658[0n]	BC-INCT1136	STDB692-14	BOLD:AAA5242	KX051423
	♂	Paratype	T. Decaens	18-Apr-2010	Brazil	Maranhao	Acailandia, REBIO do Gurupi	-4.0014, -46.8372	275	18-Apr-2010	T. Decaens	658[0n]	BC-INCT1137	STDB693-14	BOLD:AAA5242	KX051458
	♂	Paratype	T. Decaens	01-Apr-2008	Brazil	Para	Macaranduba	-4.7990, -49.3630	102	01-Apr-2008	T. Decaens	658[0n]	BC-TDMPEG0008	TDMPE008-09	BOLD:AAA5242	JN827466
	♂	Paratype	T. Decaens	01-Apr-2008	Brazil	Para	Macaranduba	-4.7990, -49.3630	102	01-Apr-2008	T. Decaens	658[0n]	BC-TDMPEG0009	TDMPE009-09	BOLD:AAA5242	JN827465
	♂	Paratype	T. Decaens	01-Apr-2008	Brazil	Para	Macaranduba	-4.8110, -49.3670	111	01-Apr-2008	T. Decaens	658[0n]	BC-TDMPEG0014	TDMPE014-09	BOLD:AAA5242	JN827464
	♂	Paratype	T. Decaens	01-Apr-2008	Brazil	Para	Macaranduba	-4.8110, -49.3670	111	01-Apr-2008	T. Decaens	658[0n]	BC-TDMPEG0301	TDMPE284-10	BOLD:AAA5242	HQ581446
	♂	Paratype	T. Decaens	01-Jun-2008	Brazil	Para	Pacaja	-3.7060, -51.0390	111	01-Jun-2008	T. Decaens	0[n]	BC-TDMPEG0667	AMAZ546-12	–	–
	♂	Paratype	T. Decaens	01-Apr-2008	Brazil	Para	Macaranduba	-4.8050, -49.3690	127	01-Apr-2008	T. Decaens	0[n]	BC-TDMPEG0743	AMAZ622-12	–	–
	♂	Paratype	T. Decaens	01-Apr-2008	Brazil	Para	Macaranduba	-4.8050, -49.3690	127	01-Apr-2008	T. Decaens	0[n]	BC-TDMPEG0744	AMAZ623-12	–	–
	♂	Paratype	T. Decaens	01-Apr-2008	Brazil	Para	Macaranduba	-4.8110, -49.3670	111	01-Apr-2008	T. Decaens	0[n]	BC-TDMPEG0918	AMAZ797-12	–	–
	♂	Paratype	T. Decaens	01-Apr-2008	Brazil	Para	Macaranduba	-4.8110, -49.3670	111	01-Apr-2008	T. Decaens	0[n]	BC-TDMPEG0919	AMAZ798-12	–	–
	♂	Paratype	T. Decaens	01-Apr-2008	Brazil	Para	Macaranduba	-4.8110, -49.3670	111	01-Apr-2008	T. Decaens	0[n]	BC-TDMPEG0920	AMAZ799-12	–	–
	♂	Paratype	T. Decaens	01-Apr-2008	Brazil	Para	Macaranduba	-4.7990, -49.3630	102	01-Apr-2008	T. Decaens	594[0n]	BC-TDMPEG0956	AMAZ835-12	BOLD:AAA5242	MT257065
	♂	Paratype	T. Decaens	01-Apr-2008	Brazil	Para	Macaranduba	-4.7990, -49.3630	102	01-Apr-2008	T. Decaens	491[0n]	BC-TDMPEG0957	AMAZ836-12	–	MT257050
	♂	Paratype	T. Decaens	01-Apr-2008	Brazil	Para	Macaranduba	-4.8040, -49.3230	116	01-Apr-2008	T. Decaens	625[2n]	BC-TDMPEG0982	AMAZ861-12	BOLD:AAA5242	MT257047
	♂	Paratype	T. Decaens	01-Apr-2008	Brazil	Para	Macaranduba	-4.8040, -49.3230	116	01-Apr-2008	T. Decaens	538[0n]	BC-TDMPEG0983	AMAZ862-12	BOLD:AAA5242	MT257063
*Automeris belizonensis*	♂	Holotype	local collector	01-Jan-2012	French Guiana	Cayenne	Kaw mountain	4.5595, -52.2067	290	01-Jan-2012	local collector	658[0n]	BC-RBP 7414	SARBE1594-13	BOLD:ACE6402	MT257076
	♀	Paratype	local collector	28-Jun-2003	French Guiana	Cayenne	Belizon	4.2697, -52.6415	80	28-Jun-2003	local collector	658[0n]	BC-EvS 1196	SAVSA1196-11	BOLD:ACE6402	JN273152
*Automeris bilinea*	♂		B. Wenczel		Venezuela	Barinas	Barinitas, Rio Calderas	8.7900, -70.4570	420		B. Wenczel	268[0n]	BC-Dec1188	STDB238-07	–	MT257049
	♂		B. Wenczel		Venezuela	Barinas	Barinitas, Rio Calderas	8.7900, -70.4570	420		B. Wenczel	658[0n]	BC-Dec1189	STDB239-07	BOLD:AAB6292	MT257046
	♀		B. Wenczel		Venezuela	Barinas	Barinitas, Rio Calderas	8.7900, -70.4570	420		B. Wenczel	658[0n]	BC-Dec1190	STDB240-07	BOLD:AAB6292	MT257072
*Automeris cinctistriga*	♂		T. Decaens, G. Lecourt	01-Dec-1991	Bolivia	La Paz	Rd Caranavi – Sta Ana	-15.5190, -67.5160	875	01-Dec-1991	T. Decaens, G. Lecourt	612[0n]	BC-Dec0707	STDA697-07	BOLD:AAA8445	MT257057
	♂		T. Decaens, G. Lecourt	01-Dec-1991	Bolivia	Beni	Rurrenabaque	-14.4640, -67.5570	380	01-Dec-1991	T. Decaens, G. Lecourt	574[0n]	BC-Dec0708	STDA698-07	BOLD:AAA8445	MT257069
	♂		T. Decaens	01-Apr-1995	Colombia	Meta	Carimagua	4.5660, -71.3330	170	01-Apr-1995	T. Decaens	635[0n]	BC-Dec0713	STDA703-07	BOLD:AAA8445	MT257054
	♀		T. Decaens	01-Apr-1995	Colombia	Meta	Carimagua	4.5660, -71.3330	170	01-Apr-1995	T. Decaens	595[0n]	BC-Dec0714	STDA704-07	BOLD:AAA8445	MT257048
	♂		T. Decaens	01-Jun-2011	French Guiana		Nouragues, Inselberg RS	4.0882, -52.6798	124	01-Jun-2011	T. Decaens	658[0n]	BC-Dec1908	STDB704-14	BOLD:AAA8445	MT257066
	♂		T. Decaens	01-Jun-2011	French Guiana		Nouragues, Inselberg RS	4.0882, -52.6798	124	01-Jun-2011	T. Decaens	658[0n]	BC-Dec1909	STDB705-14	BOLD:AAA8445	MT257062
	♂		T. Decaens	01-Jun-2011	French Guiana		Nouragues, Inselberg RS	4.0882, -52.6798	124	01-Jun-2011	T. Decaens	658[0n]	BC-Dec1910	STDB706-14	BOLD:AAA8445	MT257061
*Automeris fieldi*	♂	Paratype	H. & L. Dehez	01-Nov-1965	Colombia	Valle del Cauca	Anchicaya	3.5350, -76.8692	400	01-Nov-1965	H. & L. Dehez	407[0n]	BC-MNHN0241	STDB581-09	BOLD:AAA3341	MT257064
	♂		T. Decaens, D. Bonilla	01-Jul-2002	Colombia	Choco	San Jose del Palmar	4.9170, -76.2500	1200	01-Jul-2002	T. Decaens, D. Bonilla	658[0n]	BC-Dec0657	STDA647-07	BOLD:AAA3341	MT257058
	♂		J. Barbut et al.	11-May-2005	Costa Rica	Cartago	National Park Braulio Carillo	9.7620, -83.8820	600	11-May-2005	J. Barbut et al.	658[0n]	BC-Roug0722	SATWA741-07	BOLD:AAA3341	MT257053
*Automeris godartii*	♂		D. Herbin & M. Laguerre	02-Mar-2006	French Guiana		Dirtroad to Patagai, km. 10	5.3930, -53.1910	48	02-Mar-2006	D. Herbin & M. Laguerre	658[0n]	BC-Her0501	SDHA501-07	BOLD:AAB0970	MT257074
	♂		D. Herbin & M. Laguerre	01-Mar-2006	French Guiana		Road to St-Elie, km. 10	5.2980, -53.1500	28	01-Mar-2006	D. Herbin & M. Laguerre	658[0n]	BC-Her0550	SDHA550-07	BOLD:AAB0970	MT257055
*Automeris godartii*	♂		D. Herbin & M. Laguerre	05-Mar-2006	French Guiana		Road to Kaw, km. 32	4.5443, -52.1529	200	05-Mar-2006	D. Herbin & M. Laguerre	658[0n]	BC-Her2192	SDHC192-08	BOLD:AAB0970	MT257052
	♂		D. Herbin & M. Laguerre	02-Mar-2006	French Guiana		Dirtroad to Patagai, km. 10	5.3934, -53.1915	45	02-Mar-2006	D. Herbin & M. Laguerre	658[0n]	BC-Her2524	SDHC524-09	BOLD:AAB0970	GU703600
	♂		D. Carlot	10-Apr-1997	French Guiana		National Rd 2, km. 60	4.5170, -52.4030	64	10-Apr-1997	D. Carlot	639[0n]	BC-Roug0721	SATWA740-07	BOLD:AAB0970	MT257073
*Automeris lemensis*	♂	Paratype	C. Lemaire	17-Jun-1971	Venezuela	Bolivar	Rd El Dorado – Santa Elena, km126	5.7322, -61.4021	1350	17-Jun-1971	C. Lemaire	307[0n]	BC-MNHN0234	STDB574-09	–	MT257051
	♂	Paratype	C. Lemaire	17-Jun-1971	Venezuela	Bolivar	Rd El Dorado – Santa Elena, km126	5.7322, -61.4021	1350	17-Jun-1971	C. Lemaire	307[0n]	BC-MNHN0235	STDB575-09	–	MT257071
	♂	Paratype	C. Lemaire	17-Jun-1971	Venezuela	Bolivar	Rd El Dorado – Santa Elena, km126	5.7322, -61.4021	1350	17-Jun-1971	C. Lemaire	307[1n]	BC-MNHN0236	STDB576-09	–	MT257070
*Automeris llaneros* sp. nov.	♂	Holotype	T. Decaens, D. Bonilla	01-Aug-1999	Colombia	Casanare	Orocue	4.7943, -71.3353	150	01-Aug-1999	T. Decaens, D. Bonilla	658[0n]	BC-Dec0711	STDA701-07	BOLD:ABZ3239	MT257067
	♂	Paratype	T. Decaens, D. Bonilla	01-Jul-1999	Colombia	Meta	Carimagua	4.5716, -71.3320	170	01-Jul-1999	T. Decaens, D. Bonilla	613[0n]	BC-Dec0712	STDA702-07	BOLD:ABZ3239	MT257068
*Automeris midea*	♂		T. Decaens	07-Apr-2010	Brazil	Para	Parque Ambiental de Belem	-1.4330, -48.4110	25	07-Apr-2010	T. Decaens	658[0n]	BC-INCT0031	INCTA031-10	BOLD:ABZ7534	HQ961129
	♂		T. Decaens	07-Apr-2010	Brazil	Para	Parque Ambiental de Belem	-1.4330, -48.4110	25	07-Apr-2010	T. Decaens	658[0n]	BC-INCT0057	INCTA057-10	BOLD:ABZ7534	HQ961154
	♂		T. Decaens	11-Apr-2010	Brazil	Para	Parque Ecologico do Gunma	-1.2130, -48.2900	22	11-Apr-2010	T. Decaens	658[0n]	BC-INCT0334	INCTA334-10	BOLD:ABZ7534	HQ568029
	♂		T. Decaens	11-Apr-2010	Brazil	Para	Parque Ecologico do Gunma	-1.2130, -48.2900	22	11-Apr-2010	T. Decaens	658[0n]	BC-INCT0378	INCTA378-10	BOLD:ABZ7534	HQ568071
	♂		T. Decaens	13-Apr-2010	Brazil	Para	Reserva de Floresta da EMBRAPA	-2.1800, -48.8020	19	13-Apr-2010	T. Decaens	658[0n]	BC-INCT0551	INCTA551-10	BOLD:ABZ7534	HQ568235
	♂		T. Decaens	13-Apr-2010	Brazil	Para	Reserva de Floresta da EMBRAPA	-2.1800, -48.8020	19	13-Apr-2010	T. Decaens	658[0n]	BC-INCT0575	INCTA575-10	BOLD:ABZ7534	HQ568259
*Automeris midea*	♂		T. Decaens	18-Apr-2010	Brazil	Maranhao	Reserva Biologica Integral de Gurupi	-4.0010, -46.8370	275	18-Apr-2010	T. Decaens	658[0n]	BC-INCT1004	INCTB005-10	BOLD:ABZ7534	HQ568662
	♂		T. Decaens	08-Apr-2010	Brazil	Para	Belem, Parque Ambiental Mokambo	-1.4335, -48.4111	25	08-Apr-2010	T. Decaens	658[0n]	BC-INCT1108	STDB664-14	BOLD:ABZ7534	KX051429
*Automeris mineros* sp. nov.	♂	Holotype	G. Lecourt, D. Bonilla	01-Dec-2002	Colombia	Boyaca	Vereda Caviche	5.5756, -74.2595	1500	01-Dec-2002	G. Lecourt, D. Bonilla	658[0n]	BC-Dec0551	STDA541-07	BOLD:ABY4503	MT257075
	♀	Paratype	G. Lecourt, D. Bonilla	01-Dec-2002	Colombia	Boyaca	Vereda Caviche	5.5756, -74.2595	1500	01-Dec-2002	G. Lecourt, D. Bonilla	658[0n]	BC-Dec0547	STDA537-07	BOLD:ABY4503	MT257056
	♂	Paratype	G. Lecourt, D. Bonilla	01-Dec-2002	Colombia	Boyaca	Vereda Caviche	5.5756, -74.2595	1500	01-Dec-2002	G. Lecourt, D. Bonilla	658[0n]	BC-Dec0548	STDA538-07	BOLD:ABY4503	MT257060
	♂	Paratype	G. Lecourt, D. Bonilla	01-Dec-2002	Colombia	Boyaca	Vereda Caviche	5.5756, -74.2595	1500	01-Dec-2002	G. Lecourt, D. Bonilla	658[0n]	BC-Dec0549	STDA539-07	BOLD:ABY4503	MT257059
	♂	Paratype	G. Lecourt, D. Bonilla	01-Dec-2002	Colombia	Boyaca	Vereda Caviche	5.5756, -74.2595	1500	01-Dec-2002	G. Lecourt, D. Bonilla	373[0n]	BC-Dec0550	STDA540-07	BOLD:ABY4503	–

## References

[B1] Brechlin R Meister F (2014) Neu taxa der Gattung *Automeris* Hübner, [1819] (Lepidoptera: Saturniidae). Entomo-Satsphingia 4(1): 5–89.

[B2] Cardoso da Silva JM Rylands AB da Fonseca GAB (2005) The fate of the Amazonian Areas of Endemism. Conservation Biology 19(3): 689–694. 10.1111/j.1523-1739.2005.00705.x

[B3] Decaëns T Bonilla D Naumann S (2005) *Dirphia carimaguensis*, a new Hemileucinae from the Eastern Plains of Colombia (Lepidoptera: Saturniidae). Galathea (Nuremberg, Germany), suppl. 15: 13–21.

[B4] Decaëns T Rougerie R (2008) Description of two new species of Hemileucinae (Lepidoptera: Saturniidae) from the region of Muzo in Colombia – evidence from morphology and DNA barcodes. Zootaxa 1944: 34–52. 10.11646/zootaxa.1944.1.2

[B5] Decaëns T Rougerie R Bonilla D Ramiréz LD (2007) Contribution to the knowledge of the Saturniidae fauna of Muzo (Boyacá, Colombia), with the redescription of *Copaxa apollinairei* Lemaire, 1978 (Lepidoptera, Saturniidae). Nachrichten des Entomologischen Vereins Apollo (Frankfurt, Germany) 28(1/2): 69–75.

[B6] Felder C Felder R (1874) “Helf 4” Heterocera. Bombyces & Sphinges. In: Felder C, Felder R, Rogenhofer AF (Eds) Reise der österreichischen Fregatte Novara um die Erde in den Jahren 1857, 1858, 1859. Zoologischer Theil, Zweiter Band, Zweite Abtheilung: Lepidoptera. K.-k. Hof- und Staatsdruckerei, Vienna, plates 75–107.

[B7] Guarnizo CE Paz A Muñoz-Ortiz A Flechas SV Méndez-Narváez J Crawford AJ (2015) DNA barcoding survey of anurans across the Eastern Cordillera of Colombia and the impact of the Andes on cryptic diversity. PLoS ONE 10(5): e0127312. 10.1371/journal.pone.0127312 PMC4441516 26000447

[B8] Hajibabaei M deWaard JR Ivanova NV Ratnasingham S Dooh RT Kirk SL Mackie PM Hebert PDN (2005) Critical factors for assembling a high volume of DNA barcodes. Philosophical Transactions of the Royal Society B Biological Sciences 360: 1959–1967. 10.1098/rstb.2005.1727 PMC1609220 16214753

[B9] Hajibabaei M Janzen DH Burns JM Hallwachs W Hebert PDN (2006) DNA barcodes distinguish species of tropical Lepidoptera. Proceedings of the National Academy of Sciences of the USA 103: 968–971. 10.1073/pnas.0510466103 16418261 PMC1327734

[B10] Hebert PDN Penton EH Burns JM Janzen DH Hallwachs W (2004) Ten species in one: DNA barcoding reveals cryptic species in the Neotropical skipper butterfly *Astraptes fulgerator*. Proceedings of the National Academy of Sciences of the USA 101: 14812–14817. 10.1073/pnas.0406166101 15465915 PMC522015

[B11] Hebert PDN Ratnasingham S deWaard JR (2003) Barcoding animal life: cytochrome c oxidase subunit 1 divergences among closely related species. Proceedings of the Royal Society B Biological Sciences 270: S96–S99. 10.1098/rsbl.2003.0025 PMC1698023 12952648

[B12] Ivanova NV deWaard JR Hebert PDN (2006) An inexpensive, automation-friendly protocol for recovering high-quality DNA. Molecular Ecology Notes 6: 998–1002. 10.1111/j.1471-8286.2006.01428.x

[B13] Kitching I Rougerie R Zwick A Hamilton C St Laurent R Naumann S Ballesteros-Mejia L Kawahara A (2018) A global checklist of the Bombycoidea (Insecta: Lepidoptera). Biodiversity Data Journal 6: e22236. 10.3897/BDJ.6.e22236 PMC5904559 29674935

[B14] Lamarre GPA Decaëns T Rougerie R Barbut J Herbin D Laguerre M Thiaucourt P Martins Bonifacio M (2016) An integrative taxonomy approach unveils unknown and threatened moth species in Amazonian rainforest fragments. Insect Conservation and Diversity 9: 475–479. 10.1111/icad.12187

[B15] Lamarre GPA Mendoza I Rougerie R Decaëns T Hérault B Bénéluz F (2015) Stay out (almost) all night: contrasting responses in flight activity among tropical moth assemblages. Neotropical Entomology 44: 109–115. 10.1007/s13744-014-0264-3 26013127

[B16] Lemaire C (1969) Description d’Attacidae nouveaux de Colombie et de Panama (Lep.). Annales de la Société entomologique de France (Paris) 5: 73–94.

[B17] Lemaire C (1971) Révision du genre *Automeris* Hübner et des genres voisins. Biogéographie, éthologie, morphologie, taxonomie (Lep. Attacidae). Mémoires du Muséum national d’Histoire naturelle (Paris) (A) Zoologie 68: 1–232.

[B18] Lemaire C (1972) Description d’Attacidae (= Saturniidae) nouveaux du Venezuela et du Pérou (Lep.). Bulletin de la Société entomologique de France (Paris) 77: 29–41.

[B19] Lemaire C (2002) The Saturniidae of America (= Attacidae). Vol 4 – Hemileucinae. Goecke & Evers, Keltern, 1388 pp. [140 pls.]

[B20] Maassen JP Weyding A (1885) Beiträge zur Schmetterlingskunde. Elberfeld 5: 1–6. [pls [41–50], figs 82–126.]

[B21] Mutanen M Kivelä SM Vos RA Doorenweerd C Ratnasingham S Hausmann A Huemer P Dincă V van Nieukerken EJ Lopez-Vaamonde C Vila R Aarvik L Decaëns T Efetov KA Hebert PDN Johnsen A Karsholt O Pentinsaari M Rougerie R Segerer A Tarmann G Zahiri R Godfray C (2016) Species para- and polyphyly in COI gene trees. Systematic Biology 65(6): 1024–1040. 10.1093/sysbio/syw044 27288478 PMC5066064

[B22] Ratnasingham S Hebert PDN (2007) BOLD: The Barcode of Life Data System (http://www.barcodinglife.org). Molecular Ecology Notes 7: 355–364. 10.1111/j.1471-8286.2007.01678.x PMC1890991 18784790

[B23] Ratnasingham S Hebert PDN (2013) A DNA-based registry for all animal species: the Barcode Index Number (BIN) System. PLoS ONE 8(8): e66213. 10.1371/journal.pone.0066213 PMC3704603 23861743

[B24] Walker F (1855) List of the specimens of lepidopterous insects in the collection of the British Museum. Part VI: Lepidoptera, Heterocera. Order of the trustees, London, 1259–1507.

